# The *missing link*: TBK1 connects membrane damage sensing with autophagic response upon adenovirus entry

**DOI:** 10.1080/27694127.2022.2136604

**Published:** 2022-10-25

**Authors:** Coralie F. Daussy, Noémie Pied, Fabienne Rayne, Harald Wodrich

**Affiliations:** CNRS UMR 5234, Fundamental Microbiology and Pathogenicity, Université de Bordeaux, Bordeaux, France

**Keywords:** Adenovirus, autophagy, galectin 8, lysosomal damage, membrane damage, TBK1, virus entry

## Abstract

Most cell entry by invading pathogens involves penetration of either the plasma membrane or the endo-lysosomal compartment to reach the cytosol. This process frequently inflicts membrane damage and provokes a cellular response. This Autophagic Punctum summarizes our recent study investigating how adenovirus endosome penetration is recognized and activates macroautophagy/autophagy. Our key finding is that TBK1 (TANK binding kinase 1) has a dual role in the cell response to membrane damage; it is part of an immediate-acting membrane-damage-sensing complex and is a crucial driver of the resulting autophagic response. Thus, TBK1 is a central factor linking sensors and effectors during the cell response to membrane damage.

Cells are frequently challenged by stress of abiotic or pathogenic origin. Two well-characterized stress responses are the DNA damage and the unfolded protein response. Membrane damage has recently received much attention as an equally important stress factor in cells, which may cause intracellular release of harmful substances (e.g., lysosomal proteases) eliciting inflammation and jeopardizing cell survival. Cumulative data indicate that cells respond to membrane damage in a conserved fashion. This response probably involves a two-step process comprising the recognition of membrane damage, followed by activation of a localized form of autophagy. These processes result in the removal of damaged membranes and the control of the associated inflammatory response. Understanding how sensing of membrane damage is connected to autophagic removal at the local level has remained a challenge.

We use adenoviruses (Ads) as a model to investigate the cellular membrane damage response. Adenoviruses are non-enveloped viruses and cause extended membrane damage during endosomal escape by releasing the membrane lytic capsid protein VI. Most studies on pathogen-induced membrane damage have used invasive bacteria such as *Salmonella* Typhimurium, which replicates in vacuoles and ruptures membranes to enter the cytosol. These studies have shown that abnormal cytosolic exposure of intralumenal glycans of damaged membranes is sensed by cytosolic lectins such as LGALS3/Gal3 (galectin 3) and LGALS8/Gal8 (galectin 8). LGALS8 in particular restricts bacterial proliferation. This function involves the formation of a complex with the autophagy receptor CALCOCO2/NDP52. This complex and phosphorylation by TBK1 result in a sustained autophagy activation in response to bacterial membrane penetration. In previous work, we showed that Ad-induced membrane damage is also sensed by LGALS3 and LGALS8 and activates autophagy. However, wild type Ads are fully resistant to autophagy and secure endosomal escape by altering autophagosome maturation through a PPxY peptide motif in protein VI. Impeding the motif in a mutant virus (termed M1) renders the virus fully susceptible to autophagic degradation lowering infectivity almost tenfold and enhancing antigen presentation. Importantly, M1 infectivity is fully restored upon autophagy inactivation.

To address how the Ad-inflicted membrane damage is mechanistically linked to the ensuing autophagy response, we focused in this work on TBK1 in analogy to its role in bacterial invasion and its function in phagophore formation and stabilizing lipidated LC3 [1]. First, we demonstrated that endosome penetration during Ad entry triggers an immediate and transient TBK1 phosphorylation. Phosphorylated TBK1 accumulates specifically at the Ad membrane penetration site. Using knockout (KO)-cell models, we showed that this process is driven by LGALS8, but not LGALS3, and promotes autophagy activation.

Surprisingly, the use of additional KO-cells revealed that, despite their local recruitment, the autophagic receptors CALCOCO2, TAX1BP1 and SQSTM1/p62 (sequestosome 1) are neither required for the activation nor the recruitment of TBK1 to the membrane damage site. This observation differs significantly from what was reported for bacterial invasion, which is restricted by a TBK1-CALCOCO2 complex. TBK1 activation and recruitment also occur independently of autophagy and are not affected in *ATG5* KO cells. These results indicated that (at least) two functionally distinct complexes are activated upon Ad membrane penetration: Initially an LGALS8-dependent and TBK1-containing complex involved in sensing the membrane damage and a second complex containing autophagic receptors involved in activation of autophagy (Figure 1).

**Figure 1.**
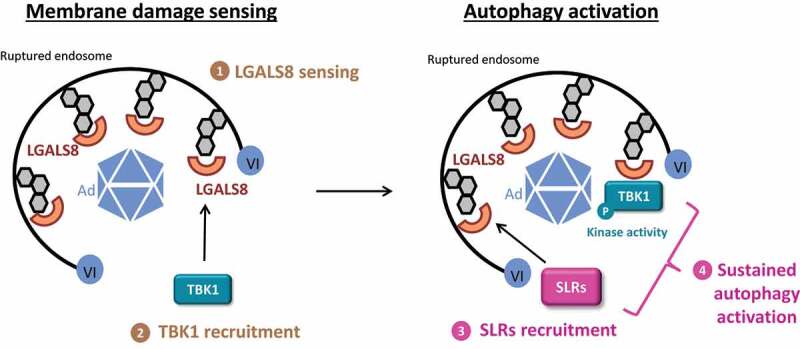
Model for TBK1 sensor and effector functions upon Ad membrane penetration. (**1**) Following protein VI release, adenovirus endosome penetration is sensed by LGALS8. (**2**) TBK1 is recruited in an LGALS8-dependent manner to the membrane damage and accumulates as an active phosphorylated form. (**3**) Autophagy receptors (sequestosome-like receptors, SLRs) are also recruited to the membrane damage site through an LGALS8-dependent process but independent of TBK1. (**4**) The presence of SLRs and active TBK1 at the membrane damage site results in sustained autophagy activation. In this context, TBK1 may phosphorylate the SLRs and/or other autophagy-related factors. Abbreviations: Ad, adenovirus; VI, protein VI; LGALS8, galectin 8; SLRs, sequestosome-like receptors.

We then addressed the role of TBK1 using both chemical and genetic TBK1 inactivation. Under both conditions, M1 infectivity was specifically (and partially) restored, showing that TBK1 is not only involved in membrane damage sensing but is also crucial for the subsequent autophagy response. Restoring M1 restriction in *TBK1* KO cells can only be achieved with catalytically active TBK1 emphasizing the importance of its kinase activity.

Our results indicated that TBK1 acts initially upstream of autophagy activation and not as its consequence but is also required downstream for sustained autophagy. Furthermore, this sensor/effector duality involving TBK1 appears kinetically and mechanistically uncoupled. We speculated that the LGALS8-TBK1 damage-sensing complex could be part of a conserved cellular response dedicated to membrane damage sensing *per se*. To follow up on this, we compared Ad endosome penetration to membrane damage induced by LLOMe, which is a specific inducer of sterile lysosomal membrane damage. LLOMe treatment also results in the activation and recruitment of TBK1 at the membrane damage site followed by LC3 lipidation and activation of autophagy. Because Ad and LLOMe damage membranes within different compartments (early endosome vs. lysosome), we compared the specificity of the cell response, depending on the origin of the damaged membrane. We found that both LGALS3 and LGALS8 are recruited to the site of lysosomal and endosomal membrane damage and that both induce TBK1 activation but at different ratios. Thus, our results may point to subtle differences in an otherwise conserved response to membrane damage owing to specific membranes.

Taken together, our study provides a comprehensive investigation into membrane damage caused by viruses. We identified TBK1 as part of a sensing machinery that uses LGALS8 to detect a wider range of membrane damage followed by a crucial role in coordinating the autophagic cell response. Furthermore, our work opens up exciting questions on the connection between virus membrane penetration vs. recognition and autophagic control through the immune kinase TBK1. Considering the importance of TBK1 in the control of other immune responses (e.g., interferon production), we might find that local TBK1 recruitment during viral membrane penetration enlists additional viral strategies to hijack cellular defenses.
